# A Comparison of Knowledge, Attitude, and Practice (KAP) Between Private- and Government-Sector Pharmacists With Regard to Psychotropic Medications in Riyadh City

**DOI:** 10.7759/cureus.54539

**Published:** 2024-02-20

**Authors:** Mohammed A Aljaffer, Saleh Alghamdi, Nuha Alkudsi, Teif Almutiri, Haya H Alanazi, Lama A Alahmadi, Bushra A Alotaibi, Norah A Almasaad

**Affiliations:** 1 Department of Psychiatry, King Saud Medical City, Riyadh, SAU; 2 Department of Psychiatry, Imam Mohammad Ibn Saud Islamic University, Riyadh, SAU; 3 College of Medicine, King Saud University, Riyadh, SAU

**Keywords:** awareness, pharmacist’s knowledge, psychiatric medications, psychopharmacology, psychotropics

## Abstract

Background

Pharmacists play a significant role in patient care, and many patients consider them to be their primary source of information regarding medications. Therefore, pharmacists must have an adequate level of knowledge about psychotropic medications. This study aims to assess and compare the levels of knowledge, attitudes, and practices regarding psychotropic medications in governmental and private-sector pharmacists in Riyadh, Saudi Arabia.

Methods

An observational cross-sectional study was conducted, which included 355 pharmacists (governmental and private sector pharmacists). Each pharmacist was interviewed and asked to answer a structured questionnaire that consisted of four sections: demographic data, knowledge, attitude, and practice regarding psychotropic medications.

Results

Our findings indicate that the overall knowledge regarding psychotropic medications among private and government-sector pharmacists is insufficient. While 282 (79.4%) had insufficient knowledge, 20.6% of pharmacists had adequate knowledge regarding psychotropic medications, and good knowledge was detected among 29.1% of government-sector pharmacists compared to 18.1% of private-sector pharmacists (P = .033). Our results also revealed that 31.5% of the pharmacists felt comfortable with their knowledge of psychotropic agents. In addition, 18.9% of the pharmacists reported that they received adequate training on psychotropic medications (12.7% of the governmental group versus 20.7% of the private-sector group; P =.048).

Conclusion

The insufficient knowledge among pharmacists regarding psychotropic medications highlights the importance of providing more training programs and educational courses to improve pharmacists’ knowledge about psychotropic medications in Saudi Arabia.

## Introduction

Globally, there has been a surge in the prevalence of mental disorders and, therefore, an increase in the consumption of psychotropic medications [1]. According to the World Health Organization (WHO), close to one billion people are living with mental disorders [2]. Moreover, a study published in 2016 indicated that one in six Americans take some kind of psychiatric medication, mostly antidepressants, which are considered the most prescribed psychotropic medication worldwide [3]. A study conducted at a primary healthcare center in Riyadh, Saudi Arabia, revealed that 28.5% of patients attending the center were diagnosed with mental disorders [4].

According to the Anatomical Therapeutic Chemical (ATC) classification, psychotropic medications are considered as belonging to one of five classes: antipsychotics, antidepressants, anxiolytics, hypnotics, and mood stabilizers. However, this classification fails to account for the recently approved psychotropic agents, which are different [5]. One limitation of the ATC classification is the potential for overlapping categories. Some medicines may have multiple therapeutic uses or may target different anatomical sites, leading to difficulties in classifying them within a single ATC code [6]. This can create ambiguity and make it challenging to accurately categorize certain medications. Additionally, the ATC classification primarily focuses on the anatomical and therapeutic properties of medicines, but it may not adequately capture other important aspects such as the mechanism of action, pharmacokinetics, or pharmacodynamics. This lack of comprehensive information may limit its usefulness in certain research or clinical settings where a more detailed classification system is required [6].

In 2020, Meraya et al. conducted a study on a sample of adults with behavioral/mental illnesses in the psychiatric outpatient clinics of five hospitals in the Jazan region of Saudi Arabia. The study indicated that most participants were prescribed antidepressants, with antipsychotics being the second most commonly prescribed medication class. The majority experienced psychotropic polypharmacy, receiving more than two psychotropic medications from the same or different class, with less than half having interclass psychotropic polypharmacy [7]. Another cross-sectional observational study was conducted at major hospitals in five regions of Saudi Arabia on patients seeking psychiatric consultation. In this study, the use of psychotropic medications was studied in order of decreasing frequency: antipsychotics, antidepressants, mood stabilizers, and antianxiety [8].

The Saudi Food and Drug Authority strictly regulates the prescription and dispensing of psychotropic medications in the country. In Saudi Arabia, such medications must be prescribed by a licensed physician. The pharmacist must not dispense drugs containing narcotics or psychotropic substances unless prescribed and approved by a physician. The prescription must be saved in the pharmacy after it has been dispensed, along with the date of drug dispensing and the number of registers in the prescription registry. The pharmacy’s seal must stamp the prescription, and the duration of these prescriptions must be specified [9]. It is worth mentioning that pharmaceutical technicians can dispense a prescription only under the supervision of a licensed pharmacist, as per the Saudi Ministry of Health regulations [10].

Most patients perceive pharmacists as a reliable source of information concerning medications, including psychotropics. Some studies have shown that the public believes that pharmacists would help them adhere to their medications and obtain the information needed about psychotropic medications and their adverse drug reactions (ADRs) [11]. Therefore, pharmacists’ knowledge regarding the medications, including their mechanisms of action and adverse reactions, plays a vital role in patient care, as pharmacists’ knowledge allows patients to be more aware of the unwanted effects of medications and safely use these medications [12]. Despite this, many recent studies have shown a lack of knowledge and awareness among community pharmacists as compared to pharmacists working in government pharmacies [13]. Moreover, some studies have shown that pharmacists generally do not deal much with psychotropic drugs [14].

The current study compares the knowledge, attitudes, and practices of government- and private-sector pharmacists in Riyadh regarding psychotropic medications. It aims to fill the knowledge gap in Saudi Arabia and provide valuable insights for pharmacists, physicians, and policymakers. The findings will help preserve patient safety and prevent errors related to psychotropic medications. The study’s findings will also emphasize the educational needs of pharmacists, if any, related to psychotropic medications.

## Materials and methods

Study design, population, and setting

This was an observational cross-sectional study conducted in Riyadh, the capital city of Saudi Arabia. The authors opted to limit the study to Riyadh for various reasons, including the ease of access to participants and the large number and variety of pharmacies in Riyadh. Moreover, the authors attempted to cover all the major areas of Riyadh (namely the Center, East, South, North, and West of Riyadh). Hence, three districts were selected randomly from Riyadh’s previously mentioned geographical areas. Data collection took place between September and October 2021. A written informed consent was obtained from all the participants before participation. Participants were informed about the title and aim of the study. They were also informed about the principal investigator’s name and contact information in case they had any questions. They were also made aware that the collected data would be anonymous, strictly confidential, and used only for research purposes. Ethical approval was given for the study by the Institutional Review Board of King Saud University (approval number: E-21-6131-CMED-305/F12).

Concerning the participants, the targeted population is the pharmacists in Riyadh. The inclusion criteria were defined as pharmacists who (a) work in a private or government pharmacy in Riyadh, (b) have a degree in pharmacy (of various levels), and (c) are Arabic or English speakers. Pharmacists who had <12 months of experience were excluded from the study.

Tool

The main tool used to conduct the study involved a questionnaire developed by the authors, comprising four sections. Data was collected by interviewing the participants using a hard copy. The questionnaire was in the English language and comprised four sections (A, B, C, and D). Section A collected the demographic data of the participants and had 10 questions. Section B focused on pharmacists’ knowledge of psychotropic medications. This second section had 30 items, subdivided into general knowledge, risk factors, and complications regarding psychotropic drugs. Section C contained questions on personal attitudes toward using psychotropic medications, and had two items. Section D included questions on the practices of pharmacists regarding psychotropic drugs, and had three items. Some of the questionnaire’s questions had “I do not know” and “I am not sure” as response options, while others did not.

Sample size

According to the Statistical Yearbook from the Ministry of Health (MOH) of Saudi Arabia, there are approximately 6,138 pharmacists in the private sector and 850 in MOH hospitals. The required sample size for this study was determined using the Steven K. Thompson formula [15]. The sample size was calculated at 95% CI and 5% of margin of error. Based on the results of applying the formula, the calculated sample size was 364 participants. Thereafter, in order to overcome the effect of possible dropout or non-completion of the questionnaire, the authors opted to add around 20% of the calculated sample size, resulting in a total of 425 participants (Note: this figure includes the calculated sample size as per the used formula and the 20% addition). Then, a pilot on 19 pharmacists from various Riyadh geographical areas was conducted to evaluate the questionnaire and perform all the necessary modifications. As no changes were made to the questionnaire based on the pilot, these participants were included in our results. 

Further into the sampling method, a multi-stage random sampling technique was used. This technique was employed to ensure equal representation of the areas included in the study. Regarding the private-sector pharmacies, the multi-stage sampling was based on areas of Riyadh (Center, East, South, North, and West) as the first stage. In the second stage, three districts were selected randomly from each geographical area. In the third stage, within each area, 65 pharmacists were chosen. As for government pharmacists, the same technique was used; we targeted 10 government hospitals, two from each geographical area (Center, East, South, North, and West). Ten pharmacists were selected from each hospital. The ratio of the government-sector to private-sector pharmacists was based on the fact that there are more private pharmacists than government ones in Riyadh.

Data analysis

Data were analyzed using the statistical software IBM SPSS Statistics for Windows, Version 22.0 (Released 2013; IBM Corp., Armonk, New York, United States). Descriptive statistics (mean, standard deviation, frequencies, and percentages) were used to describe the quantitative and categorical variables. Univariate analysis was carried out using student’s t-test for independent samples, one-way analysis of variance, and post-hoc test for quantitative outcome variables to compare the mean values in relation to the categorical study variables, which have two or more options. Pearson’s chi-squared test was used to measure the relationship between the categorical study and outcome variables, and the exact probability test was used for small frequency distributions. ORs were calculated to measure the association. If the chi-squared test (for a two × two table) was not applicable, Fisher’s exact test was used. All statistical analyses were done using a two-tailed test with a p-value <0.05 and 95% confidence intervals to report the statistical significance and precision of the results.

The data were collected, analyzed, and weighted as follows: (1) for knowledge items, each correct answer was scored one point, and then the total of the points was calculated; (2) a pharmacist with a score of less than 60% (i.e., less than 18 points out of the total score) was considered to have poor knowledge, while pharmacists were considered to have good knowledge if they had a score of 60% or more.

## Results

Out of the 425 pharmacists who were interviewed and asked to fill out the questionnaire, 355 (83.5%) successfully completed it while the remaining did not. The demographic information of the pharmacists included in the study is shown in Table 1.

**Table 1:** Descriptive data of study pharmacists in Riyadh city, Saudi Arabia SR: Saudi Riyal

Personal data	Number	Percentage
Age in years		
24-30	195	54.9%
31-40	121	34.1%
41-50	33	9.3%
51-60	6	1.7%
Gender		
Male	266	74.9%
Female	89	25.1%
Pharmacist’s education		
Technician	8	2.3%
BSc Pharm (Bachelor of Science in Pharmacy)	224	63.1%
MSc (Master of Science in Pharmacy)	28	7.9%
PharmD (Doctor of Pharmacy)	95	26.8%
Nationality		
Saudi	133	37.5%
Non-Saudi	222	62.5%
Pharmacy’s ownership (Practice setting)		
Government	79	22.3%
Private (Chain)	243	68.5%
Private (Hospital)	20	5.6%
Private (Single)	13	3.7%
Experience in years		
< 5	138	38.9%
5-10	122	34.4%
> 10	95	26.8%
Monthly income		
< 5000 SR	49	13.8%
5000-10000 SR	211	59.4%
11000-20000 SR	87	24.5%
> 20000 SR	8	2.3%
Work hours daily		
≤ 8 hours	95	26.8%
> 8 hours	260	73.2%
Have you taken a course on psychotropic medications out of your pharmacy degree program?		
Yes	73	20.6%
No	282	79.4%

Table 2 illustrates the pharmacists’ knowledge regarding psychotropic medications. Of the pharmacists, 25.4% answered “false” for the statement “I would not prescribe selective serotonin reuptake inhibitors (SSRIs) to a patient with a history of seizure disorder” (36.7% of governmental pharmacists versus 22.1% of the private sector pharmacists) with recorded statistical significance (P = .004). Fluoxetine was identified as the antidepressant with the longest half-life by 47.3% of the pharmacists (59.5% for governmental pharmacists versus 43.8% for private sector pharmacists; P = .047). Of the pharmacists, 57.2% agreed that antiepileptic drugs can be used as mood stabilizers (67.1% for the governmental group and 54.3% for the private group, respectively; P = .001). Additionally, 79.7% agreed that benzodiazepine can lead to physical and emotional dependence (65.8% of governmental pharmacists versus 83.7% of private-sector pharmacists; P = .001). The statement “antipsychotics are a good choice for a sleep disorder” was refused by 52.4% of the pharmacists (41.8% of the governmental group versus 55.4% of the private sector group; P = .004). Answering the question “Which of the following drugs could help with sexual dysfunction?”, 28.2% of the pharmacists answered “bupropion” (38% of governmental pharmacists versus 25.4% of private pharmacists; P = .002). Also, 23.7% of the pharmacists answered the question related to the mechanism of action of Haldol to be as that of ziprasidone (25.3% of governmental versus 23.2% of the private sector; P= .043). There were no significant differences among the pharmacists in all other general knowledge items. 

**Table 2:** Comparison of general knowledge regarding psychotropic medications between private and governmental pharmacists ^P^ Pearson X^2^ test; ^$^ Exact probability test; ^*^P < 0.05 (significant), Choices that have been made bold are the correct answers. TCA: tricyclic antidepressant; SSRI: selective serotonin reuptake inhibitor; MAO: monoamine oxidase; SNRI: serotonin and norepinephrine reuptake inhibitor

Questions	Choices	Total		Work Pharmacy				p-value
				Government		Private		
		Number	Percentage	Number	Percentage	Number	Percentage	
Which of the following benzodiazepines has the highest risk of addiction?	Short-acting	139	39.1	40	50.7	99	35.9	0.055
	Medium acting	39	11.0	8	10.1	31	11.2	
	Long-acting	177	49.9	31	39.2	146	52.9	
The recommended 1st line treatment for anxiety disorders is currently benzodiazepines.	True	103	29.0	16	20.2	87	31.5	0.136
	False	197	55.5	48	60.8	149	54.0	
	Not sure	55	15.5	15	19.0	40	14.5	
Ophthalmic effects (Blurred vision) are more prominent with	TCAs	165	46.5	31	39.2	134	48.6	0.191
	SSRIs	105	29.6	31	39.2	74	26.8	
	MAOs	67	18.9	14	17.7	53	19.2	
	SNRIs	18	5.1	3	3.8	15	5.4	
Orthostatic hypotension is a well-known adverse effect of	TCAs	131	36.9	29	36.7	102	37.0	0.427
	SSRIs	73	20.6	21	26.6	52	18.8	
	MAOs	115	32.4	23	29.1	92	33.3	
	SNRIs	36	10.1	6	7.6	30	10.9	
There is a connection between long-term intake of antipsychotic medications and the prevalence of falls in the elderly	True	190	53.5	42	53.2	148	53.6	0.938
	False	41	11.5	10	12.7	31	11.2	
	Do not know	124	34.9	27	34.2	97	35.1	
I would not prescribe SSRI to a patient with a history of seizure disorder	True	187	52.7	29	36.7	158	57.2	0.004*
	False	90	25.4	29	36.7	61	22.1	
	Don’t know	78	22.0	21	26.6	57	20.7	
SSRI is my first-choice psychotropic medication for depression in most cases	True	270	76.1	64	81.0	206	74.6	0.190
	False	49	13.8	6	7.6	43	15.6	
	Don’t know	36	10.1	9	11.4	27	9.8	
Is it safe to prescribe SSRI and MAO antidepressants at the same time?	Yes	31	8.7	8	10.1	23	8.3	0.363
	No	260	73.2	53	67.1	207	75.0	
	Not sure	64	18.0	18	22.8	46	16.7	
Which of the following antidepressants has the longest half-life?	Fluoxetine	168	47.3	47	59.5	121	43.8	0.047*
	Paroxetine	80	22.5	13	16.5	67	24.3	
	Escitalopram	107	30.1	19	24.1	88	31.9	
Can antiepileptic drugs be used as mood stabilizers?	Yes	203	57.2	53	67.1	150	54.3	0.001*
	No	94	26.5	7	8.9	87	31.5	
	Not sure	58	16.3	19	24.1	39	14.1	
Benzodiazepine can lead to physical and emotional dependence	True	283	79.7	52	65.8	231	83.7	0.001*
	False	34	9.6	11	13.9	23	8.3	
	Don’t know	38	10.7	16	20.3	22	8.0	
Olanzapine is associated with anticholinergic side effects	True	226	63.7	53	67.1	173	62.7	0.740
	False	63	17.7	12	15.2	51	18.5	
	Don’t know	66	18.6	14	17.7	52	18.8	
Clozapine is the first line to treat psychosis	True	130	36.6	26	32.9	104		
	False	161	45.4	33	41.8	128	37.7	
	Don’t know	64	18.0	20	25.3	44	15.9	
Antipsychotics are a good choice for a sleep disorder	True	122	34.4	27	34.2	95	34.4	0.004*
	False	186	52.4	33	41.8	153	55.4	
	Don’t know	47	13.2	19	24.1	28	10.1	
Which of the following drugs could help with sexual dysfunction?	Bupropion	100	28.2	30	38.0	70	25.4	
	Reboxetine	74	20.8	21	26.6	53	19.2	
	Escitalopram	137	38.6	16	20.3	121	43.8	
	Trazodone	44	12.4	12	15.2	32	11.6	
Which drug in the SSRI group has a half-life of 2-4 days?	Fluoxetine	122	34.4	32	40.5	90	32.6	0.097
	Paroxetine	48	13.5	14	17.7	34	12.3	
	Escitalopram	59	16.6	7	8.9	52	18.8	
	Sertraline	126	35.5	26	32.9	100	36.2	
Rivastigmine belongs to	Cholinesterase inhibitors	248	69.9	55	69.6	193	69.9	0.297^$^
	Inorganic ions	17	4.8	4	5.1	13	4.7	
	MAOs	42	11.8	6	7.6	36	13.0	
	Butyrophenones	17	4.8	3	3.8	14	5.1	
	Stimulants	31	8.7	11	13.9	20	7.2	
Which of the following is not an antidepressant?	Pimozide	239	67.3	58	73.4	181	65.6	0.479
	Trazodone	50	14.1	10	12.7	40	14.5	
	Amitriptyline	47	13.2	9	11.4	38	13.8	
	Fluoxetine	19	5.4	2	2.5	17	6.2	
The mechanism of action of Haldol (haloperidol) is	Ziprasidone	84	23.7	20	25.3	64	23.2	0.043*
	Serotonin reuptake inhibitor	67	18.9	9	11.4	58	21.0	
	MAO-B inhibitor	135	38.0	39	49.4	96	34.8	
	Benzodiazepine	69	19.4	11	13.9	58	21.0	
Lithium is used in psychiatry to treat	Acute mania	246	69.3	56	70.9	190	68.8	0.065
	Hysteria	39	11.0	13	16.5	26	9.4	
	Phobia	20	5.6	5	6.3	15	5.4	
	Acute organic brain syndrome	50	14.1	5	6.3	45	16.3	
What is the best alternative for Fluoxetine?	None	43	12.1	11	13.9	32	11.6	0.890
	Paroxetine	170	47.9	38	48.1	132	47.8	
	Escitalopram	89	25.1	20	25.3	69	25.0	
	Sertraline	53	14.9	10	12.7	43	15.6	
What is the best alternative for Risperidone?	None	82	23.1	19	24.1	63	22.8	0.141
	Olanzapine	124	34.9	35	44.3	89	32.2	
	Clonidine	17	4.8	2	2.5	15	5.4	
	Ziprasidone	132	37.2	23	29.1	109	39.5	

As for risk factors regarding psychotropic medications (Table 3), 50.1% of the pharmacists agreed that bupropion is not recommended for patients with epilepsy (62% of the governmental group versus 46.7% of the private sector group; P = .039). Of the pharmacists, 17.7% answered that antipsychotics do not have a high risk of addiction with long-term use (25.3% vs. 15.6%, respectively; P = .001). Considering complications (Table 3), 51% of the pharmacists agreed that venlafaxine is associated with increases in blood pressure (62% of governmental pharmacists versus 47.8% of private pharmacists; P = .029). Also, 43.1% of the pharmacists knew that olanzapine is a medication that is accompanied by a higher risk of weight gain (53.2% vs. 40.2%, respectively; P = .018). Concerning the overall knowledge about psychotropic medications among private and governmental sector pharmacists, 73 (20.6%) pharmacists had a good level of knowledge, while 282 (79.4%) had an insufficient level of knowledge.

**Table 3:** Knowledge on risk factors and complications regarding psychotropic medications among private and governmental pharmacists ^P^ Pearson X^2^ test; ^$^ Exact probability test; ^*^P < 0.05 (significant) Choices that have been made bold are the correct answers. SSRI: selective serotonin reuptake inhibitor

			Total		Work Pharmacy				P-value
					Government		Private		
			Number	Percentage	Number	Percentage	Number	Percentage	
Risk factors	Bupropion is not recommended in patients with epilepsy	True	178	50.1	49	62.0	129	46.7	.039*
		False	79	22.3	11	13.9	68	24.6	
		Don’t know	98	27.6	19	24.1	79	28.6	
	Antipsychotics have high risk of addiction with long use	True	250	70.4	42	53.2	208	75.4	.001*
		False	63	17.7	20	25.3	43	15.6	
		Don’t know	42	11.8	17	21.5	25	9.1	
	Extra-pyramidal symptoms are associated with	Antipsychotics	198	55.8	52	65.8	146	52.9	.089
		Antidepressants	69	19.4	14	17.7	55	19.9	
		Benzodiazepine	88	24.8	13	16.5	75	27.2	
	Serotonin syndrome is associated with	Antidepressants	237	66.8	54	68.4	183	66.3	.258
		Antipsychotics	73	20.6	19	24.1	54	19.6	
		Benzodiazepine	45	12.7	6	7.6	39	14.1	
Complications	Patients on SSRIs may develop a sense of emotional detachment	True	214	60.3	48	60.8	166	60.1	.828
		False	57	16.1	14	17.7	43	15.6	
		Don’t know	84	23.7	17	21.5	67	24.3	
	Venlafaxine is associated with increases in blood pressure	True	181	51.0	49	62.0	132	47.8	.029*
		False	75	21.1	9	11.4	66	23.9	
		Don’t know	99	27.9	21	26.6	78	28.3	
	This medication has a higher risk of weight gain	Olanzapine	153	43.1	42	53.2	111	40.2	.018*
		Bupropion	80	22.5	9	11.4	71	25.7	
		Fluoxetine	122	34.4	28	35.4	94	34.1	
	Paroxetine has a higher risk of causing sexual dysfunction in comparison with other SSRIs	True	195	54.9	44	55.7	151	54.7	.077
		False	82	23.1	12	15.2	70	25.4	
		Don’t know	78	22.0	23	29.1	55	19.9	

Table 4 demonstrates pharmacists’ attitudes and practices regarding psychotropic medications. Of the pharmacists, 31.5% felt comfortable with their knowledge of psychotropic agents. In addition, 18.9% of the pharmacists reported that they received adequate training on psychotropic medications (12.7% of the governmental group versus 20.7% of the private group; P =.048). As for practice regarding psychotropic medications, 66.5% of the pharmacists always rechecked and asked about chronic diseases, such as diabetes, hypertension, heart and kidney diseases, and epilepsy, before dispensing psychotropic medications. Only 15.8% of pharmacists reported that they suggested a different psychotropic medication from that prescribed by the doctor (19% of the governmental group versus 14.9% of the private group; P = .001). Considering the prompt “the number of psychiatric patients who use your pharmacy in one month,” 47.9% of the pharmacists reported “less than 10 patients” (40.5% vs. 50%, respectively), while 18.3% reported “more than 40 patients” (32.9% vs. 14.1, respectively; P = .003). 

**Table 4:** Attitude and practice regarding psychotropic medications among private and governmental pharmacists ^P^ Pearson X^2^ test; ^$^ Exact probability test; ^*^P < 0.05 (significant)

Domain	Items	Total		Work pharmacy				p-value
				Government		Private		
		Number	Percentage	Number	Percentage	Number	Percentage	
Attitude	Do you feel comfortable with your knowledge of psychotropic agents?							.111
	Yes	112	31.5	18	22.8	94	34.1	
	No	166	46.8	39	49.4	127	46.0	
	Not sure	77	21.7	22	27.8	55	19.9	
	Do you get adequate training on psychotropic medications?							.048*
	Yes	67	18.9	10	12.7	57	20.7	
	No	233	65.6	51	64.6	182	65.9	
	Not sure	55	15.5	18	22.8	37	13.4	
Practice	As a pharmacist, I always recheck and ask about some chronic diseases such as (diabetes, hypertension, heart diseases, kidney diseases, epilepsy) before dispensing psychotropic medications.							.227
	Yes	236	66.5	52	65.8	184	66.7	
	Sometimes	86	24.2	23	29.1	63	22.8	
	No	33	9.3	4	5.1	29	10.5	
	Have you ever suggested a different psychotropic medication from that prescribed by the doctor?							.001*
	Yes	56	15.8	15	19.0	41	14.9	
	Sometimes	47	13.2	21	26.6	26	9.4	
	No	252	71.0	43	54.4	209	75.7	
	Approximately how many psychiatric patients are currently using your pharmacy in one month’s time?							.003*^$^
	< 10	170	47.9	32	40.5	138	50.0	
	11-20	59	16.6	12	15.2	47	17.0	
	21-30	36	10.1	4	5.1	32	11.6	
	31-40	25	7.0	5	6.3	20	7.2	
	> 40	65	18.3	26	32.9	39	14.1	

The distribution of pharmacists’ overall knowledge regarding psychotropic medications is demonstrated in Table 5. A good level of knowledge was detected among 31.5% of female pharmacists in comparison to 16.9% of male pharmacists, with recorded statistical significance (P = .003). Also, 30.5% of pharmacists who had a Doctor of Pharmacy degree (PharmD) had a good knowledge level compared to 16.1% of those with only a bachelor’s degree (P = .034). Good knowledge regarding psychotropic medications was detected among 29.1% of governmental sector pharmacists compared to 18.1% of private sector pharmacists (P = .033). In addition, 28.4% of pharmacists who worked for less than eight hours daily had a good knowledge level compared to 17.7% of those who worked for more than eight hours (P = .027).

**Table 5:** Distribution of pharmacists’ overall knowledge regarding psychotropic medications ^P^ Pearson X^2^ test; ^$^ Exact probability test; ^*^P < 0.05 (significant) SR: Saudi Riyal

	Overall knowledge level				p-value
	Poor		Good		
	Number	Percentage	Number	Percentage	
Age in years					.189^$^
24-30	153	78.5	42	21.5	
31-40	101	83.5	20	16.5	
41-50	25	75.8	8	24.2	
51-60	3	50.0	3	50.0	
Gender					.003*
Male	221	83.1	45	16.9	
Female	61	68.5	28	31.5	
Pharmacist’s education					.034*
Technician	6	75.0	2	25.0	
BSc Pharm (Bachelor of Science in Pharmacy)	188	83.9	36	16.1	
MSc (Master of Science in Pharmacy)	22	78.6	6	21.4	
PharmD (Doctor of Pharmacy)	66	69.5	29	30.5	
Nationality					.071
Saudi	99	74.4	34	25.6	
Non-Saudi	183	82.4	39	17.6	
Work pharmacy					.033*
Government	56	70.9	23	29.1	
Private	226	81.9	50	18.1	
Experience in years					.302
< 5	104	75.4	34	24.6	
5-10	101	82.8	21	17.2	
> 10	77	81.1	18	18.9	
Monthly income					.698
< 5000 SR	37	75.5	12	24.5	
5000-10000 SR	172	81.5	39	18.5	
11000-20000 SR	67	77.0	20	23.0	
> 20000 SR	6	75.0	2	25.0	
Work hours daily					.027*
≤ 8 hours	68	71.6	27	28.4	
> 8 hours	214	82.3	46	17.7	
Have you taken a course on psychotropic medications out of your pharmacy degree program?					.742
Yes	59	80.8	14	19.2	
No	223	79.1	59	20.9	

## Discussion

Our results revealed that most pharmacists from both sectors had poor knowledge regarding psychotropic medications, with better results seen in government-sector pharmacists. Our finding is consistent with the results of a cross-sectional study conducted in Ethiopia regarding pharmacists’ knowledge and practice of issues related to the use of psychotropic medications in the elderly. More than half of the pharmacists in the study had limited knowledge of such medications [16]. Another study conducted in Palestine regarding pharmacists’ knowledge of women’s issues with epilepsy indicated that pharmacists had poor knowledge regarding psychotropic medications [17]. Other studies have also revealed a limited level of knowledge and awareness among pharmacists regarding ADRs [18]. Such a low level of understanding could compromise patient safety and raise the possibility of mistakes when dispensing psychotropic medications [19].

Our results showed a statistically significant difference in the monthly income between the private and government sectors, indicating a higher income among government pharmacists. Our results are consistent with another published study about pharmacists’ psychotropic medication knowledge in Ethiopia. The study revealed that respondents with higher monthly incomes had significantly higher levels of knowledge than those with lower monthly incomes. There was also a statistically significant difference in the number of working hours in each sector. Almost half of the government-sector pharmacists work less than eight hours a day compared to only a minority of the private-sector pharmacists. Concerning the relationship between workload, the quality of care, and pharmacists’ satisfaction, it has been well documented in several studies that not having enough time to ﬁnish work was associated with lower levels of job satisfaction, burnout, as well as poor practice [20,21]. Moreover, researchers have concluded that exploring subjective measures of workload is very important for improving pharmacists’ practice and performance [22].

Regarding the number of psychiatric patients visiting the pharmacy per month, about one-third of the government-sector pharmacists in our study reported that more than 40 psychiatric patients use their pharmacy in one month compared to significantly fewer numbers in the private sector. These results indicate that government-sector pharmacists deal with psychotropic medications more often. This finding is consistent with a study conducted in Riyadh in 2006 regarding community pharmacists’ attitudes toward mental illness and providing pharmaceutical care for mentally ill patients. The results of this study showed that pharmacists who have had more contact with mentally ill patients have more positive attitudes toward the provision of pharmaceutical care to such patients [22].

Education levels in the two sectors we studied also differed, with more pharmacists with PharmD degrees working in the government sector than in the private sector. Our results also illustrate that more pharmacists with a PharmD have good level of knowledge than pharmacists with any other degree in pharmacy. This finding was similar to that reported in another study about Jordanian pharmacists’ knowledge of issues related to the use of psychotropic medications. Researchers found that the scientific degree was the most frequent variable associated with knowledge of pharmacists and that pharmacists who held a PharmD degree had the highest percentage of correct answers, followed by pharmacists with a master’s degree, and then pharmacists with a bachelor’s degree [23]. Similar findings from a study conducted in India regarding the evaluation of knowledge, attitude, and practices of Indian pharmacists pertaining to ADR reporting suggest that post-graduate pharmacists (M. Pharm, PharmD, Ph.D.) responded significantly more than pharmacists with other qualifications [24].

Although the difference in the years of experience of pharmacists in the two sectors in our study was not statistically significant, our results revealed that pharmacists who had less than five years of experience had better knowledge regarding psychotropic medications. Consistent with our finding, in another study, pharmacists with a job experience of three to four years had a greater understanding and better knowledge of the appropriate use of drugs as compared to pharmacists with 9-10 years of experience [25]. Another cross-sectional study that was conducted in Lebanon on the knowledge of pharmacists and parents towards antibiotic use in pediatrics revealed a negative association between years of experience and sound knowledge of antibiotic use. This study revealed that poor knowledge was associated with a greater number of years of experience [26]. However, these findings differ from those of another study on the role of community pharmacists in patients’ counseling and health education concerning diabetes mellitus type II. This study revealed that those with more than five years of work experience and a postgraduate qualification had higher mean practice scores than those with less than five years of work experience and only a diploma qualification [27]. Another study revealed that pharmacists who had five years of experience or more had a better level of knowledge and practice on issues related to using psychotropic medication in elderly people in Ethiopia [16]. Furthermore, a study on pharmacists’ knowledge of women’s issues with epilepsy revealed no significant associations with age, degree, number of years in practice, or receiving training on epilepsy and AEDs during their degree program [28].

Pharmacists are at the frontline when it comes to handling and dispensing medications; therefore, it is important to ensure that they have adequate training and education and feel comfortable with their level of knowledge to ensure safe practice. Our results indicate that most pharmacists from both sectors do not feel comfortable with their level of knowledge regarding psychotropic medications. However, there were statistically significant differences found between the two sectors, with a higher number of pharmacists in the private sector responding that they had adequate training in psychotropic medications than those in the government sector. The difference observed in our results regarding training programs could be attributed to different factors, including demographic variables, age, nationality, and the universities from which pharmacists graduated. In line with our findings, the results of a survey on pharmacists’ perceptions of the adequacy of their training for addressing mental health-related medication issues indicated that, in a group of experienced pharmacists, many did not feel comfortable counseling patients on mental health-related medications [29]. This finding is also consistent with another study regarding community pharmacists’ attitudes towards mental illness conducted in Riyadh, which found that pharmacists reported feeling uncomfortable counseling and solving drug-related problems for patients with mental illness [23,30]. Furthermore, several studies revealed that pharmacists were significantly more confident and comfortable in providing services to consumers with cardiovascular disease than with mental illnesses [30,31]. Another study conducted in the United Arab Emirates on how to provide mental healthcare through community pharmacies revealed that pharmacists described feelings of emotional discomfort when dealing with patients with mental illnesses. Researchers found that pharmacists cited two main challenges in providing care for mentally ill patients: the emotional distress felt by them and a lack of awareness and training. Moreover, pharmacists highlighted that training in effective communication with mentally ill patients is often not provided in pharmacy programs [30].

Further indicating the importance of training programs, a study in Jordan on pharmacists’ knowledge of issues related to the use of psychotropic medications showed that pharmacists who had received training in psychiatry were two to three times more likely to answer questions correctly than those who had not [23]. Another study conducted in Germany regarding drug-drug interactions in psychiatry revealed that clinical pharmacists can contribute to higher drug therapy safety in psychiatric wards, emphasizing the importance of providing psychotropic medication courses to pharmacists. The study’s results revealed that after the pharmacists had joined the ward and educated staff about drug interactions, the relevant drug interactions were significantly reduced [32]. Moreover, a study regarding pharmacists’ perceptions toward continuing education revealed that Saudi pharmacists showed a great desire for continuing education, believing that it would greatly enhance their professional performance [18].

Our analysis of gender differences revealed that female pharmacists had higher scores in good knowledge in comparison to their male counterparts, with the difference observed being statistically significant. In line with our finding, a study in Palestine on pharmacists’ knowledge of issues related to using psychotropic medications in older people showed that female pharmacists had better knowledge of issues related to using psychotropic medications than their male peers [17]. In another study on pharmacists’ knowledge and level of involvement in relation to diabetes mellitus type II, similar findings were reported, indicating that female pharmacists had higher mean knowledge than male pharmacists in the management of the disease [28]. A similar finding was reported in another study, revealing that female pharmacists had an increased knowledge concerning antibiotic use in children compared to male pharmacists [13]. In contrast, findings from a study conducted in Pakistan concerning the knowledge, attitude, and practices of community pharmacists regarding COVID-19 revealed that female pharmacists had lower odds of adequate knowledge compared to their male counterparts [25]. Nonetheless, according to a study of students’ knowledge of and attitudes toward psychotropic drugs, gender was not statistically related to their knowledge of psychotropic medication [33].

Although our study addresses an important topic that has not been adequately studied in Saudi Arabia, it has certain limitations. One is the length of the questionnaire used, which may have had some influence on the response rate or at least led some participants not to complete it fully. Another limitation is that this study was conducted in only one city in Saudi Arabia, i.e. Riyadh, which makes it difficult to generalize the results. Therefore, more multicenter studies with larger sample sizes are warranted.

## Conclusions

Based on the findings of the current study, it is evident that there is a general lack of comprehensive understanding among both private- and government-sector pharmacists when it comes to psychotropic medications. However, comparatively better scores were observed among pharmacists working within the government sector. This highlights the need for additional educational opportunities to bridge the knowledge gap surrounding psychotropic drugs, specifically tailored courses, and workshops aimed at equipping pharmacists in Saudi Arabia with the necessary expertise in this domain.

## Appendices

Questionnaire

Dear participant, Thank you for agreeing to take part in this survey. The purpose of this survey is to measure the pharmacists’ awareness, knowledge, and attitude about Psychotropic medications. You are being asked to take part in a research study that aims to measure the awareness of pharmacists in the private and governmental sectors in Riyadh city regarding psychotropic medications,  when participating in the study, you will have to answer 40 questions through an interview with the data-collector, all answers will be entered into Google form for more accuracy. - A multiple-choice 40 questions should be completed to achieve the purpose of participation. - If you decide to participate in this study, you may withdraw from your participation at any time without penalty. - All responses are completely anonymous and all information will be used for scientific purposes. - There is no cost for participating in this study and no rewards. - All your answers will be kept confidential.

**Table 6:** Questionnaire survey TCA: tricyclic antidepressant; SSRI: selective serotonin reuptake inhibitor; MAO: monoamine oxidase; SNRI: serotonin and norepinephrine reuptake inhibitor; SR: Sudi Riyal

Demographics (personal info)		
Nationality		Saudi
		Non-Saudi
Age (years)		24-30
		31-40
		41-50
		51-60
Gender		Male
		Female
Pharmacist’s education (Degree)		MSc (master of science in pharmacy)
		PhD (doctor of philosophy)
		PharmD (Doctor of Pharmacy)
		BSc Pharm (bachelor of science in pharmacy)
		Technician
Pharmacy’s ownership (Practice setting)		Government
		Private (Chain)
		Private (Single)
		Private (Hospital)
Experience (years in practice)		< 5
		5-10
		>10
Income (salary)		< 5000 SAR
		5000-10,000 SAR
		>10,000 SAR
Working Hours		≤ 8
		> 8
Have you taken a course on psychotropic medications out of your pharmacy degree program?		Yes
		No
Personal Knowledge About the Use of Psychotropic Medications		
Which of the following Benzodiazepines has the highest risk of addiction?		Long-acting
		Medium acting
		Short-acting
The recommended treatment for anxiety disorders is currently benzodiazepines.		True
		False
		I’m not sure
Ophthalmic effects (Blurred vision ) are more prominent with which of the following?		TCAs
		SSRIs
		MAOs
		SNRIs
Orthostatic hypotension is a well-known adverse effect of?		SSRIs
		TCAs
		SNRIs
		MAOs
There is a connection between long-term (3 months or above) intake of antipsychotic medications and the prevalence of falls in the elderly.		True
		False
		I don’t know
I would not prescribe SSRI to a patient with a history of seizure disorder.		True
		False
		I don’t know
SSRI is my first choice for depression in most cases.		True
		False
		I don’t know
Is it safe to prescribe SSRI and MAO antidepressants at the same time?		No
		Yes
		I’m not sure
Which of the following antidepressants has the longest half-life?		Paroxetine
		Escitalopram
		Fluoxetine
Can antiepileptic drugs be used as mood stabilizers?		No
		Yes
		I’m not sure
Benzodiazepine can lead to physical and emotional dependence.		True
		False
		I don’t know
Olanzapine is associated with anticholinergic side effects.		True
		false
		I don’t know
Clozapine is the first line to treat psychosis.		True
		False
		I don’t know
Antipsychotics are good choice for sleep disorder.		True
		False
		I don’t know
Which drug could help with sexual dysfunction?		Bupropion
		Reboxetine
		Escitalopram
		Trazodone
Which drug in the SSRI group has a half-life of 2-4 days?		Paroxetine
		Escitalopram
		Sertraline
		Fluoxetine
Rivastigmine belongs to which of the following?		Inorganic ions
		MAOs
		Butyrophenones
		Stimulants
		Cholinesterase inhibitors
Which of the following is not an antidepressant?		Trazodone
		Amitriptyline
		Flouxetine
		Pimozide
The mechanism of action of Haldol is?		Serotonin reuptake inhibitor
		MAO-B inhibitor
		Ziprasidone
		Benzodiazepine
Lithium is used in psychiatry to treat?		Hysteria
		Phobia
		Acute organic brain syndrome Indications of lithium in psychiatry
		Acute mania
What is the best alternative for Fluoxetine?		Paroxetine
		Escitalopram
		Sertraline
		None
What is the best alternative for Risperidone?		Olanzapine
		Clonidine
		Ziprasidone
		None
Risk factors		
Bupropion is not recommended in patients with epilepsy.		True
		False
		I don’t know
Antipsychotics have high risk of addiction with long use.		True
		False
		I don’t know
Extra-pyramidal symptoms are associated with?		Antipsychotics
		Antidepressants
		Benzodiazepine
Serotonin syndrome is associated with?		Antipsychotics
		Antidepressants
		Benzodiazepine
Complications		
Patients on SSRIs may develop a sense of emotional detachment	True	
	False	
	I don’t know	
Venlafaxine is associated with increases in blood pressure.	True	
	False	
	I don’t know	
This medication has a higher risk of weight gain	Olanzapine	
	Bupropion	
	Fluoxetine	
Paroxetine has higher risk of causing sexual dysfunction in comparison with other SSRIs	True	
	False	
	I don’t know	
Personal Attitudes About the Use of Psychotropic Medications		
Do you feel comfortable with your knowledge of psychotropic agents?	Yes	
	No	
	I’m not sure	
Do you get adequate training on psychotropic medications?	Yes	
	No	
	I’m not sure	
Personal Practice About the Use of Psychotropic Medications		
As a pharmacist, I always recheck and ask about some chronic diseases such as (diabetes, hypertension, heart diseases, kidney diseases, epilepsy) before dispensing psychotropic medications	Yes	
	No	
	Sometimes	
Have you ever suggested a different psychotropic medication from that prescribed by the doctor?	Yes	
	No	
	Sometimes	
Approximately how many psychiatric patients are currently using your pharmacy in one month’s time?	10 or less	
	11-20	
	21-30	
	31-40	
	More than 40	
